# Supplementary material for: Oral prodrug of remdesivir parent GS-441524 is efficacious against SARS-CoV-2 in ferrets
Source: Nat Commun. 2021 Nov 5;12:6415. doi: 10.1038/s41467-021-26760-4 (PMC8571282; doi:10.1038/s41467-021-26760-4)
Supplement: Supplementary file 1 — Supplementary Information [file 41467_2021_26760_MOESM1_ESM.pdf]

Oral prodrug of remdesivir parent GS-441524 is efficacious against SARS-CoV-2 in ferrets

Robert M. Cox[1], Josef D. Wolf[1], Carolin M. Lieber[1], Julien Sourimant[1], Michelle J Lin[2], Darius Babusis[3], Venice Du Pont[3], Julie Chan[3], Kim T. Barrett[3], Diane Lye[3], Rao Kalla[3], Kwon Chun[3], Richard L. Mackman[3], Chengjin Ye[4], Tomas Cihlar[3], Luis Martinez-Sobrido[4], Alexander L. Greninger[2], John P. Bilello[3], Richard K. Plemper[1]*

[1]Center for Translational Antiviral Research, Institute for Biomedical Sciences, Georgia State University, Atlanta, GA, USA

[2]Virology Division, Department of Laboratory Medicine and Pathology, University of Washington, Seattle, WA

[3]Gilead Sciences Inc., Foster City, CA, USA

[4]Texas Biomedical Research Institute, San Antonio, TX, USA.

Supplementary Information

Supplementary Table 1. Antiviral potency and cytotoxicity
Supplementary Table 2. Single dose pharmacokinetic parameters of GS-441524 following administration of either intravenous GS-441524 or remdesivir or oral GS-621763 in ferrets
Supplementary Table 3. GS-441524 and its metabolite concentrations in ferret lung tissue
Supplementary Table 4. Primers used for qPCR of ferret samples.
Supplementary Table 5. SRA and GenBank accession codes
Supplementary Figure 1. Remdesivir cytotoxicity
Supplementary Figure 2. Clinical signs in source and contact animals infected with BZ/2021

Supplementary Table 1. Antiviral potency and cytotoxicity.

virus	host cells	GS-621763		GS-441524		remdesivir	
		EC_{50} [μM]	CC_{50} [μM]	EC_{50} [μM]	CC_{50} [μM]	EC_{50} [μM]	CC_{50} [μM]
WA1/2020-nano luciferase	A549-ACE2	0.72 ± 0.32[a]	>47[b]	1.4 ± 0.9[c]	>50[b]	0.08 ± 0.03[d]	17.3 ± 4.9[c]
WA1/2020[e]	VeroE6	0.73	>100	0.68	>100	n.d.	>100
CA/2020 (α)[e]	VeroE6	0.21	>100	0.11	>100	n.d.	>100
SA/2020 (β)[e]	VeroE6	0.11	>100	0.34	>100	n.d.	>100
BZ/2021 (γ)[e]	VeroE6	0.22	>100	0.55	>100	n.d.	>100
n.a.[e]	HEp-2	n.a.	79.4	n.a.	>100	n.a.	45.12
n.a.[e]	VeroE6	n.a.	>100	n.a.	>100	n.a.	>100
n.a.[e]	BHK-21	n.a.	>100	n.a.	>100	n.a.	>100
n.a.[e]	HCT-8	n.a.	74.6	n.a.	>100	n.a.	36.4
n.a.[e]	"F2" HAE	n.a.	43.8	n.a.	>100	n.a.	85.5
n.a.[e]	"F3" HAE	n.a.	43.8	n.a.	>100	n.a.	104.4
n.a.[e]	"M2" HAE	n.a.	39.7	n.a.	>100	n.a.	101.9
n.a.[e]	"M6" HAE	n.a.	92.3	n.a.	>100	n.a.	>100
n.a.[e]	"DF2" HAE	n.a.	86	n.a.	>100	n.a.	>33

[a]mean ± SD (n=4); data represent mean of four independent experiments, each with technical duplicates.

[b]mean (n=6); data represent mean of six independent experiments, each with technical triplicates or quadruplicates.

[c]mean ± SD (n=8); data represent mean of eight independent experiments, each with technical duplicates.

[d]mean ± SD (n=12); data represent mean of twelve independent experiments, each with technical duplicates.

[e]mean (n=3); all other data represent mean of three independent experiments.

Supplementary Table 2. Single dose pharmacokinetic parameters of GS-441524 following administration of either intravenous GS-441524 or remdesivir or oral GS-621763 in ferrets.

compound	route	dose [mg/kg]	$t_{1/2}$ [hours]	IV – CL [L/hours/kg] PO – T_{max} [hours]	C_{max} [µM]	AUC_{last} [µM.h]	F [%]
GS-441524	i.v.	20	3.4	0.86	54.2	81.1	n/a
remdesivir	i.v.	10	6.09	n/a	2.81	18.2	n/a
GS-621763[a]	p.o.	30	2.68 ± 0.15	4.0 ± 3.5	15.8 ± 4.7	80.8 ± 14.6	115 ± 21

[a]approximately 10 nM GS-621763 transiently observed in first two hours

Supplementary Table 3. GS-441524 and its metabolite concentrations in ferret lung tissue.

compound	route	dose	lung GS-443902 [nmol/g]	lung total nuc [nmol/g]
GS-441524	i.v.	20 mg kg^{-1}	0.53 ± 0.10	0.66 ± 0.21
remdesivir	i.v.	10 mg kg^{-1}	1.28[a]	2.96
GS-621763	p.o.	30 mg kg^{-1}	0.30 ± 0.19	0.88 ± 0.13

[a]one lung from remdesivir i.v. dosing was BLQ for all metabolites

Supplementary Table 4. Primers used for qPCR of ferret samples.

primer ID	sequence
nCoV_IP2-12669_Fw	5'-ATGAGCTTAGTCCTGTTG-3'
nCoV_IP2-12759_Rv	5'-CTCCCTTTGTTGTGTTGT-3'
nCoV_IP2-12696 probe	[5']Fam-AGATGTCTTGTGCTGCCGGTA-[3']BHQ-1
nCoV_IP4-14146_Rv	5'-CTGGTCAAGGTTAATATAGG-3'

Supplementary Table 5. SRA and GenBank accession codes.

Strain	Sample Type	SRA Accession	GenBank Accession
WA1/2020	Inoculum	SRR14883752	MZ433205
WA1/2020	vehicle #1	SRR14883763	MZ433206
WA1/2020	vehicle #2	SRR14883762	MZ433207
WA1/2020	vehicle #3	SRR14883751	MZ433208
WA1/2020	vehicle #4	SRR14883749	MZ433209
WA1/2020	treated #1	SRR14883748	MZ433210
WA1/2020	treated #2	SRR14883747	MZ433211
WA1/2020	treated #3	SRR14883746	MZ433212
WA1/2020	treated #4	SRR14883745	MZ433213
BZ/2021	inoculum	SRR14883750	MZ433225
BZ/2021	vehicle #1	SRR14883744	MZ433214
BZ/2021	vehicle #2	SRR14883743	MZ433215
BZ/2021	vehicle #3	SRR14883761	MZ433216
BZ/2021	vehicle #4	SRR14883760	MZ433217
BZ/2021	treated #1	SRR14883759	MZ433218
BZ/2021	treated #2	SRR14883758	MZ433219
BZ/2021	treated #3	SRR14883757	MZ433220
BZ/2021	treated #4	SRR14883756	MZ433221
BZ/2021	contact vehicle #1	SRR14883755	MZ433222
BZ/2021	contact vehicle #2	SRR14883752	MZ433205
BZ/2021	contact vehicle #3	SRR14883763	MZ433206

Supplementary Figure 1

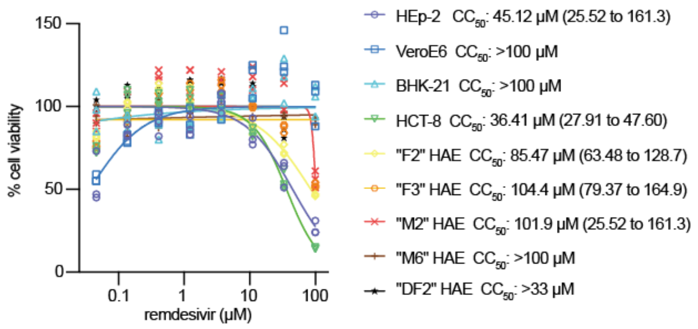

Supplementary Fig. 1. Remdesivir cytotoxicity. *In vitro* cytotoxicity profile of remdesivir on HEp-2, VeroE6, BHK-21, HCT-8 and the panel of primary HAE cells ("F2", "F3", "M2", "M6", "DF2") cells examined in (Fig. 2b-e). Symbols represent individual biological repeats (n=3), lines depict non-linear regression models.

Supplementary Figure 2

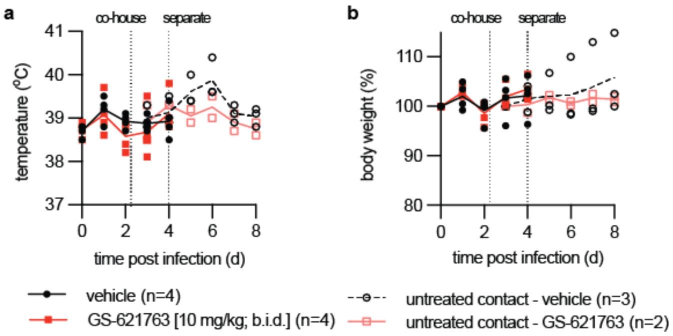

Supplementary Fig. 2. Clinical signs in source and contact animals infected with BZ/2021. a, Temperature measurements collected once daily. **b,** Body weight measured once daily.
