# Supplementary material for: Oral prodrug of remdesivir parent GS-441524 is efficacious against SARS-CoV-2 in ferrets
Source: Nat Commun. 2021 Nov 5;12:6415. doi: 10.1038/s41467-021-26760-4 (PMC8571282; doi:10.1038/s41467-021-26760-4)
Supplement: Supplementary file 3 — Description of Additional Supplementary Files [file 41467_2021_26760_MOESM3_ESM.pdf]

Description of Additional Supplementary Files

File Name: Supplementary Data 1
Description: Bukeh plots for all VOC γ metagenomic analyses.

File Name: Supplementary Data 2
Description: Bukeh plots for all WA1/2020 metagenomic analyses.

File Name: Supplementary Data 3
Description: Source data of results shown in figures 1-4, Supplementary figures 1-2, and extended data tables 1-3.

File Name: Supplementary Data 4
Description: Statistical analyses of results shown in figures 2-4.
