# Supplementary material for: Oral prodrug of remdesivir parent GS-441524 is efficacious against SARS-CoV-2 in ferrets
Source: Nat Commun. 2021 Nov 5;12:6415. doi: 10.1038/s41467-021-26760-4 (PMC8571282; doi:10.1038/s41467-021-26760-4)
Supplement: Supplementary file 5 — Supplementary Data 2 [file 41467_2021_26760_MOESM5_ESM.html]

WA2020
