# Supplementary material for: Oral prodrug of remdesivir parent GS-441524 is efficacious against SARS-CoV-2 in ferrets
Source: Nat Commun. 2021 Nov 5;12:6415. doi: 10.1038/s41467-021-26760-4 (PMC8571282; doi:10.1038/s41467-021-26760-4)
Supplement: Supplementary file 8 — Reporting Summary [file 41467_2021_26760_MOESM8_ESM.pdf]

nature research

Corresponding author(s): Richard K Plemper

Last updated by author(s): Oct 16, 2021

Reporting Summary

Nature Research wishes to improve the reproducibility of the work that we publish. This form provides structure for consistency and transparency in reporting. For further information on Nature Research policies, see our Editorial Policies and the Editorial Policy Checklist.

Statistics

For all statistical analyses, confirm that the following items are present in the figure legend, table legend, main text, or Methods section.

n/a	Confirmed	
☐	☒	The exact sample size (*n*) for each experimental group/condition, given as a discrete number and unit of measurement
☐	☒	A statement on whether measurements were taken from distinct samples or whether the same sample was measured repeatedly
☐	☒	The statistical test(s) used AND whether they are one- or two-sided<br>*Only common tests should be described solely by name; describe more complex techniques in the Methods section.*
☒	☐	A description of all covariates tested
☐	☒	A description of any assumptions or corrections, such as tests of normality and adjustment for multiple comparisons
☐	☒	A full description of the statistical parameters including central tendency (e.g. means) or other basic estimates (e.g. regression coefficient) AND variation (e.g. standard deviation) or associated estimates of uncertainty (e.g. confidence intervals)
☐	☒	For null hypothesis testing, the test statistic (e.g. F, t, r) with confidence intervals, effect sizes, degrees of freedom and P value noted<br>*Give P values as exact values whenever suitable.*
☒	☐	For Bayesian analysis, information on the choice of priors and Markov chain Monte Carlo settings
☒	☐	For hierarchical and complex designs, identification of the appropriate level for tests and full reporting of outcomes
☒	☐	Estimates of effect sizes (e.g. Cohen's *d*, Pearson's *r*), indicating how they were calculated

Our web collection on statistics for biologists contains articles on many of the points above.

Software and code

Policy information about availability of computer code

Data collection	Excel (Versions 16.42, 16.43, and 16.48; Microsoft) and Numbers (version 10.1; Apple) used for most data collection. Reverse transcription qPCR data was collected using an Applied Biosystems StepOnePlus PCR system using the StepOnePlus software (Version 2.1; Applied Biosystems). All PrestoBlue cytotoxicity measurements were obtained using a Biotek H1 synergy plate reader using Gen5 software (version 2.06.10). For reporter virus activity and cytotoxicity assays in A549-hACE2 cells, signals were measured using an Envision microplate reader (Perkin Elmer).
Data analysis	Statistical analyses were performed in the Prism (GraphPad) software package (versions, 8.0, 9.0.1, and 9.1.0). Figures were composed using Adobe Illustrator (version CS6). Reverse transcription qPCR data was analyzed using the StepOnePlus software (Version 2.1; Applied Biosystems). Pharmacokinetic parameters were calculated using Phoenix WinNonlin (version 8.2, Certara) and concentration-time profiles generated using Prism (version 8, GraphPad). SARS-CoV-2 next-generation sequencing analysis was performed using , Sequenase (v2.0), Trimmomatic (v0.39), bwa (version 0.7.17), Picard (version 2.18.15), VarScan (version 2.3), Annovar (2018Apr16 version), and Longitudinal Analysis of Viral Alleles (LAVA; version 1), available at (https://github.com/michellejlin/lava). Four-parameter variable slope regression modeling and statistical analyses were performed in the Prism (GraphPad) software package (Version 9.1.0). Power analyses were carried out using GPower 3.1 (University of Duesseldorf).

For manuscripts utilizing custom algorithms or software that are central to the research but not yet described in published literature, software must be made available to editors and reviewers. We strongly encourage code deposition in a community repository (e.g. GitHub). See the Nature Research guidelines for submitting code & software for further information.

Data

Policy information about availability of data

All manuscripts must include a data availability statement. This statement should provide the following information, where applicable:

- Accession codes, unique identifiers, or web links for publicly available datasets
- A list of figures that have associated raw data
- A description of any restrictions on data availability

All data generated or analyzed during this study are included in this published article (and its supplementary information files). Numerical source data for figures 1-4 and Supplementary figures 1-2 are provided with the paper in Supplementary dataset 3. Summaries of statistical analyses are provided in Supplementary Dataset 4. All next-generation sequencing data is publicly available on SRA (BioProject PRJNA740065). All sequences were deposited in Genbank under the following accession numbers: MZ433205, MZ433206, MZ433207, MZ433208, MZ433209, MZ433210, MZ433211, MZ433212, MZ433213, MZ433214, MZ433215, MZ433216, MZ433217, MZ433218, MZ433219, MZ433220, MZ433221, MZ433222, MZ433223, MZ433224, and MZ433225.

Field-specific reporting

Please select the one below that is the best fit for your research. If you are not sure, read the appropriate sections before making your selection.

[x] Life sciences   [ ] Behavioural & social sciences   [ ] Ecological, evolutionary & environmental sciences

For a reference copy of the document with all sections, see nature.com/documents/nr-reporting-summary-flat.pdf

Life sciences study design

All studies must disclose on these points even when the disclosure is negative.

Sample size	Appropriate sample sizes were determined using the Resource Equation and Power analysis. Unless otherwise noted, at least three samples were used for each group.
Data exclusions	No data was excluded from this manuscript.
Replication	All experimental data were reliably produced; the number of independent biological repeats and, when applicable, technical repeats is specified for each experiment. At least three biological (independent) replicates were performed for all in vitro experiments experiments. In vivo efficacy used at least three biological (independent) replicates per experiment. All attempts at replication of results were successful.
Randomization	Animals and samples were randomly sorted into experimental groups.
Blinding	The investigators were not blinded to group allocation for data collection and analysis for any experiment performed in this study due to size of the research group with clearance for experimentation under high biocontainment conditions required for work with live SARS-CoV-2. Ferrets are large animals that cannot be handled by a single investigator and resources available did not allow involvement of additional personnel that would have been required for blinding.

Reporting for specific materials, systems and methods

We require information from authors about some types of materials, experimental systems and methods used in many studies. Here, indicate whether each material, system or method listed is relevant to your study. If you are not sure if a list item applies to your research, read the appropriate section before selecting a response.

Materials & experimental systems

n/a	Involved in the study	
x		Antibodies
	x	Eukaryotic cell lines
x		Palaeontology and archaeology
	x	Animals and other organisms
x		Human research participants
x		Clinical data
x		Dual use research of concern

Methods

n/a	Involved in the study	
x		ChIP-seq
x		Flow cytometry
x		MRI-based neuroimaging

Eukaryotic cell lines

Policy information about cell lines

Cell line source(s)	African green monkey kidney cells VeroE6 (ATCC®, cat# CRL-1586™)<br>Human Lung Carcinoma Cells (A549) Expressing Human Angiotensin-Converting Enzyme 2 (BEI, #NR-53821)<br>human lung adenocarcinoma epithelial cells Calu-3 (ATCC® HTB-55™)<br>human epithelial/HeLa contaminant HEp-2 cells (ATCC®, cat# CCL-23™)<br>baby hamster kidney cells BHK-21 (ATCC®, cat# CCL-10™)<br>human lung adenocarcinoma epithelial cells A549 expressing hACE2 (Mossel et al. PMID 15731278)<br>Human epithelial colon adenocarcinoma HCT-8 cells (ATCC® cat# CCL-244™ lot# 70036111)<br>Human Bronchial Tracheal Epithelial cells (HBTEC) were derived from the following donors:<br>"F2" from a 29-year old Caucasian female (Lifeline, cat# FC-0035, lot# 5101)<br>"F3" from a 42-year old Caucasian female (Lonza, cat# CC-2540S, lot# 0000519670)<br>"M2" from a 40-year old Caucasian male (Lonza, cat# CC-2540S, lot# 0000667744)<br>"M6" from a 48-year old Caucasian male (Lonza, cat# CC-2540S, lot# 0000544414).<br>Diseased (Asthma) Human Bronchial Epithelial (DHBE) cells "DF2" were from a 55-year old Caucasian female (Lonza, cat# 00194911S, lot# 0000534647)
Authentication	Cells were authenticated by the supplier or by morphological appearance and susceptibility to virus infection including SARS-CoV-2.
Mycoplasma contamination	Cell lines were confirmed mycoplasma-negative when obtained from the supplier, followed by preparation and cryo-preservation of master and working stocks. Individual working stocks were replaced every three months. All cell lines in use in the laboratory were routinely retested for mycoplasma contamination in 6-months intervals and tested negative.
Commonly misidentified lines<br>(See ICLAC register)	HEp-2 cells are indicated in the ICLAC database of commonly misidentified cell lines. Use of these cells was necessary, since they are highly permissive for respiratory syncytial virus, which is susceptible to inhibition by remdesivir, and the parent drug GS-441524

Animals and other organisms

Policy information about studies involving animals; ARRIVE guidelines recommended for reporting animal research

Laboratory animals	This study used female ferrets (Mustela putorius furo), family mustelids, genus mustela, 6-10 months of age.
Wild animals	This study did not involve wild animals.
Field-collected samples	This study did not involve field-collected samples.
Ethics oversight	Experiments with SARS-CoV-2 involving ferrets were approved by the Georgia State Institutional Animal Care and Use Committee under protocol A20031. All experiments using infectious SARS-CoV-2 were approved by the Georgia State Institutional Biosafety Committee under protocol B20016.

Note that full information on the approval of the study protocol must also be provided in the manuscript.
